# On the Molecular Level Cavitation in Soft Gelatin Hydrogel

**DOI:** 10.1038/s41598-020-66591-9

**Published:** 2020-06-15

**Authors:** KAH Al Mahmud, Fuad Hasan, Md Ishak Khan, Ashfaq Adnan

**Affiliations:** Mechanical and Aerospace Engineering, University of Texas at Arlington, Arlington, Texas, USA

**Keywords:** Biomedical materials, Atomic and molecular physics, Mechanical engineering, Physics

## Abstract

We have studied the molecular level cavitation mechanisms and bubble growth kinetics in soft gelatin hydrogel and water. The apparent difference in cavitation threshold pressure between that generates in pure water and that in gelatin hydrogel is considered. Gelatin, which is derived from collagen, is frequently used as a brain simulant material. In liquid, cavitation bubble is created when surrounding pressure drops below the saturation vapor pressure. In principle, a cavitation bubble should continue to grow as long as tensile pressure continues to increase in the system. In our study, using molecular dynamics simulation, we have investigated the pressure requirement for a nanoscale cavitation to grow in water and gel. First, we have modeled a gel like structure with a preexisting bubble of 5 nm radius. A control model containing a 5 nm bubble in pure water is also created. Then, we have applied hydrostatic tensile pressure at two different expansion rates in the gel and water models. The results show that a gel-like structure requires higher pressure for the cavitation to grow, and both gel and water models exhibit strain rate effect on the cavitation threshold pressure. We have also found that the cavitation collapse time is dominated by the viscosity of the medium.

## Introduction

Cavitation initiates in a liquid or soft material when pressure drops below the saturated vapor pressure. Soft materials like gelatins are formed when interconnected collagen or collagen-like molecules form a cellular network in bulk water. A slight changes in the applied pressure can collapse a cavitation bubble and induce high pressure to the adjacent cells to initiate damage. Damaging capabilities of collapsing cavitation bubbles are well known. For example, severe erosion damage to propeller blades of a sea vehicle can occur due to high-energy cavitation bubble collapse over the blade surface. By the same mechanism, implosions in fluid-filled biological systems such as soft tissues or brain may experience significant damage^1^.

**Figure 1:**
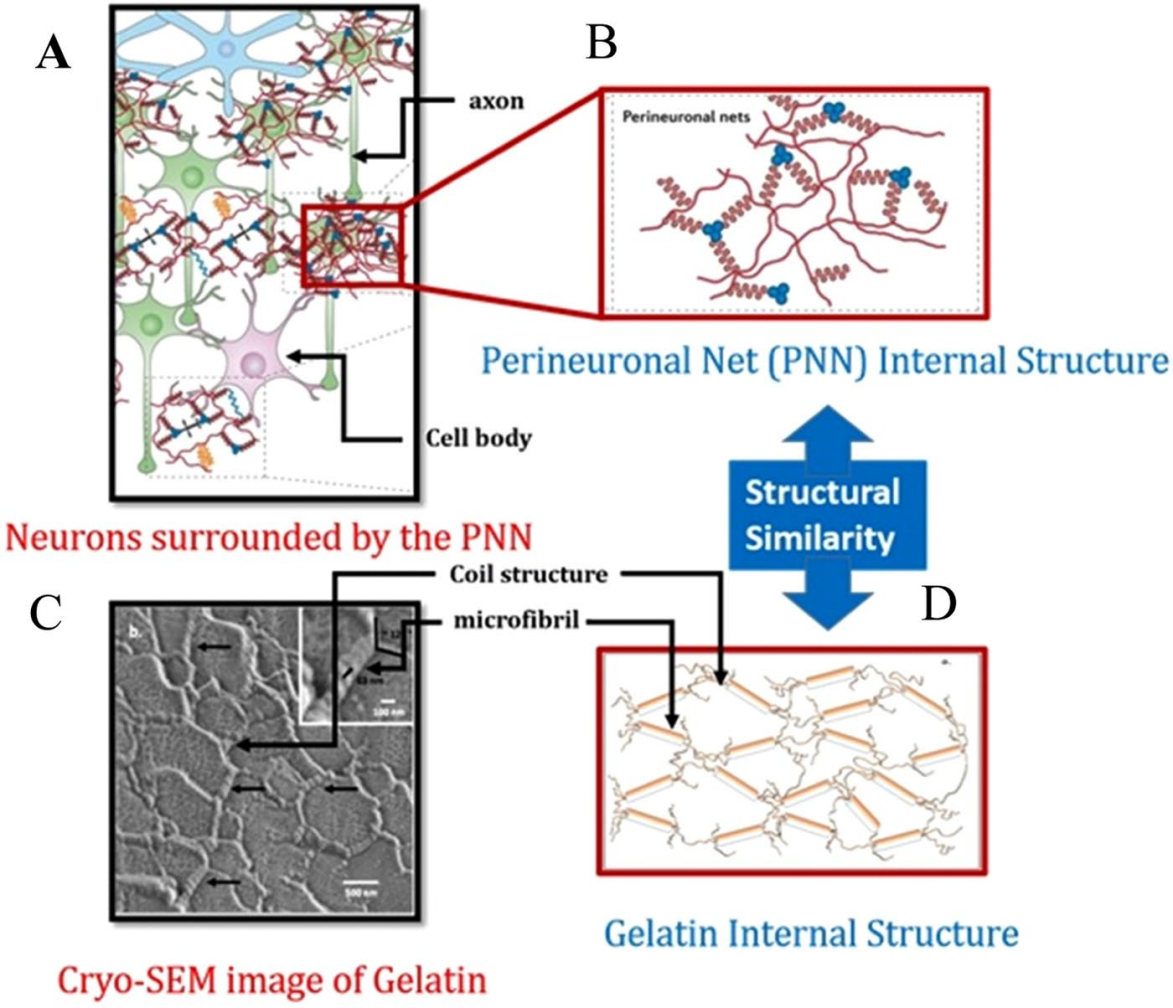
(**A**) Schematic illustration of neurons surrounded by the perinuronal net (PNN) of extra cellular matrix (ECM) (adapted from Fig. 1 of^5^), (**B**) Internal structure of PNN, (**C**) Cryo-SEM image of 10% (w/w) gelatin gel with 0.5% mg/ml cross linking agent (adapted from Fig. 3 of ref. ^9^), (**D**) schematic of gelatin network model (adapted from Fig. 4 of ref. ^9^). The structural similarities between gelatin network (in **D**) and PNN (in **B**) are noticeable.

Recently, understanding the cavitation mechanisms in soft materials gained attention due to its potential implication in biomedical related applications^2–5^. Soft materials such as gelatin hydrogels are widely used as a brain simulant material^6–8^. In addition, the stiffness and other physical properties of gelatin gel can be tuned by changing the gel concentration in aqueous solution. Recently, reasonable structural similarities are observed between the gel network and brain’s perineuronal net (PNN)^9^ (λure 1A,B,D). A high resolution cryo-SEM image shows that a gelatin microstructure consists of both crystalline and coil like amorphous part (Fig. 1C), where the crystalline part is made of collagen-D periodic microfibrillar structure^10^, and the coil part, found only at the network junction, is composed of amorphous collagen chains.

Cavitation mechanisms is widely studied for water^11–13^ and fairly well understood. However, cavitation mechanism in biological soft matter is not explored well. Cavitation in gel is complicated to analyze because biological soft materials are labile and semisolid in nature, and exhibit nonlinear elastic deformation^14^. Moreover, interfacial tension between water and gel network may play an important role, which, in turn, may affect the cavitation mechanics. At the molecular scale, the affinity of amino acid residue with water is complex because different residues have different types of interactions with water, namely hydrophobic, hydrophilic and charged (positively or negatively). In the solution of gelatin, the crystalline collagen may interact with three types of water molecules - (a) free bulk water, (b) bound water that interacts with the gelatin surface, and (c) structural water that are trapped within gelatin molecules stabilize the triple helix^15^. In the continuum scale, surface tension and viscosity of gelatin gel are not constant, rather, they depend on gelation time and concentration^16^. Due to these structural and chemical instabilities and time variant material properties, accurately predicting cavitation pressure for gel be very challenging.

Few studies are available on cavitation in soft viscoelastic materials such as gelatin^7^, polyacrylamide hydrogels^17^, etc. Wonmo Kang *et al*.^7^ observed that cavitation pressure sharply rises in 1% gelatin solution when compared with pure water (Fig. 3(a) of ref. ^7^). It has been hypothesized that the rise in cavitation threshold in gel is due to the higher surface tension, preexistence thermal residual stress, and nonspherical bubble formation in gel. Other parameters such as interfacial tension, stiffness of gel fibril, bubble surrounding environment properties (e.g. density, viscosity) during growth and collapse may also play critical role.

The critical cavitation pressure depends on several factors such as temperature, viscosity, surface tension, and cavitation time or strain rate. Among them, surface tension and temperature play major role. The other two factors - viscosity and strain rate - impart insignificant impact on critical cavitation pressure^18^. Very few studies have explored the effect of strain rate on the critical cavitation pressure of liquid water^12,19^, and soft biological materials^20^. Interestingly, Stan *et al*.^12^ found that at the higher strain rate the bubble growth velocity is not sufficient to relax the tension, which causes more than three times higher negative pressure for bubble growth at ten times higher strain rate. On the other hand, Fisher^18^ proposed that strain rate effect is negligible. He showed using an theoretical model that, for the homogenous cavitation time ratio of ${10}^{33}\,({10}^{-15}\,{\rm{s}}\,{\rm{the}}\,{\rm{atomistic}}\,{\rm{vibration}}\,{\rm{range}}\,{\rm{and}}\,{10}^{18}\,{\rm{s}}\,{\rm{the}}\,{\rm{age}}\,{\rm{of}}\,{\rm{universe}})$, critical cavitation pressure changes only 1.58 times. This claim does not necessarily depict that the change in the critical cavitation pressure would proportionately vary over all ranges of strain rates, and heterogenous cavitation may have different mechanics. Moreover, in this study, the viscous effect on the critical cavitation pressure has been ignored. Viscosity arises from both intermolecular and intramolecular forces, electrostatic forces and frictional forces due to mechanical entanglement among the gel chains^21^. Details of the contributing factors (surface tension, rigidity, viscosity) that affect the cavitation critical pressure have been described in Appendix 4 of supplementary materials.

The aim of this work is to gain fundamental insights of molecular level bubble growth and collapse mechanism in gel and water. First, we have modeled a gelatin-like system based on a cryo-SEM image of gelatin. A preexisting bubble of 5 nm radius is then placed at the center of this model. Then, we have applied hydrostatic tensile pressure at two different expansion rates in gel and water models. The threshold pressures for cavitation bubble growth in water and gel have been estimated. The mechanisms of bubble growth in water and gel have been studied in detail.

## Results and discussion

At first, we have studied the heterogenous cavitation bubble growth mechanisms in a gel-like and pure water system. The critical pressure for bubble growth has been estimated. Next, we have studied the bubble stabilization kinetics. We have also analyzed the strain rate effect on bubble growth pressure. Finally, we have examined the collapsing mechanism of cavitation bubble in gel and water.

### Cavitation bubble growth

It is known that when a pure liquid, such as water, is allowed to expand at a constant rate, at some point, a tiny void (or many voids at the same time) will form inside the pure liquid. The equilibrium is maintained through the balance of surface tension and the applied tensile (expansion) pressure. In the cavitation community, the tensile pressure is often referred as negative pressure. In the absence of vapor pressure, the equilibrium equation for stable bubble is^22^

1 $$\frac{2{S}_{{\rm{liq}}}}{R}+{{P}_{{\rm{cav}}}|}_{{\rm{liq}}}=0$$

where, ${{{\rm{P}}}_{cav}|}_{liq}$ = required far field pressure for stable bubble in a pure liquid, ${{\rm{S}}}_{liq}$ = surface tension of the liquid, and R = stable bubble radius.

we argue that, the presence of network in gel consumes additional strain energy that must be overcome for the growth of the cavitation. As such, the required cavitation pressure in gel ${{{\rm{P}}}_{cav}|}_{gel}$ would be higher than $\,{{{\rm{P}}}_{cav}|}_{liq}$. Qualitatively, this relation can be written as

2 $$\frac{2{S}_{{\rm{gel}}}}{R}+{{P}_{{\rm{cav}}}|}_{{\rm{gel}}}=0$$

where, ${{{\rm{P}}}_{cav}|}_{gel}=$ ${{{\rm{P}}}_{cav}|}_{liq}+{{{\rm{P}}}_{cav}|}_{network},\,$ where, ${{{\rm{P}}}_{cav}|}_{network}$ is the pressure equivalent of the additional strain energy (bending, tensile and torsoinal)^23^ associated with the gel network deformation and S_gel_, is the surface tension of gel.

**Figure 2:**
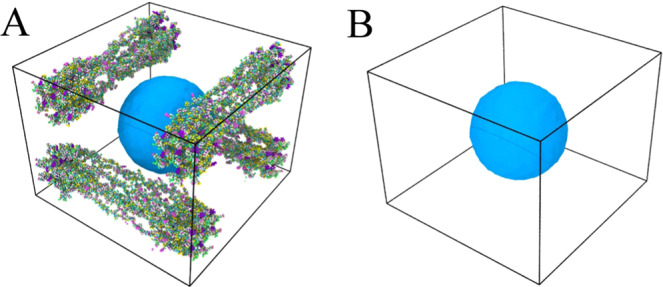
Models for cavitation study (**A**) Gel like model (gel like fibril is modeled using UCSF chimera [‘https://www.cgl.ucsf.edu/chimera/’ version 1.13.1]^37^) (**B**) Water (The Images are visualized by Ovito [‘https://www.ovito.org/’ version 3.0.0-dev]^32^).

Once the bubble is nucleated, it then continues to grow as long as the condition for bubble growth is maintained. Here, we have studied the molecular level cavitation growth phenomena in water and gel like structures. The gel model is built on four collagen fibrils that are arranged in a network forming architecture and immersed in water molecules. The fibrils are placed at the top and bottom surface of the model (Fig. 2A), whereas Fig. 2B shows the water model. The gel like structure has approximately 10.4% (w/w) collagen molecule. To simulate cavitation growth, a uniform hydrostatic tensile (negative pressure) stress is induced to the system by increasing the system volume at a strain rate of X = 6.7 × 10^9^ s^−1^ and 2X = 1.35 × 10^10^ s^−1^, respectively. The application of the uniform tensile pressure implies overall increase in the system volume. Note that system volume ($${V}_{system}$$) includes water volume ($${V}_{water})$$, the bubble volume ($${V}_{bubble}=\frac{4}{3}\pi {R}^{3}$$) and gel network $$({V}_{network}$$), as written below

$${V}_{system}={V}_{water}+\,{V}_{network}+{V}_{bubble}\left(=\frac{4}{3}\pi {R}^{3}\right)$$

**Figure 3:**
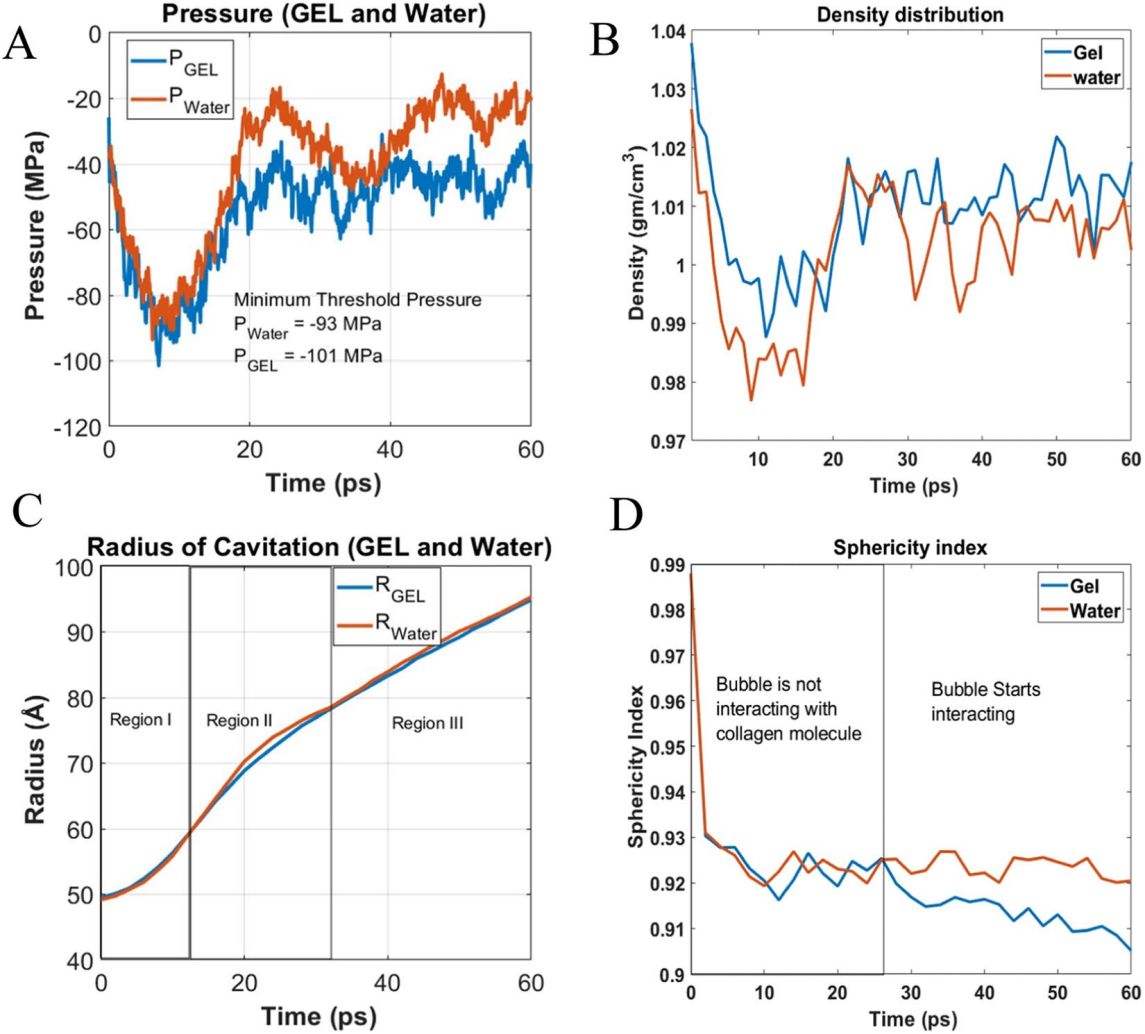
Bubble growth phenomena in water and gel like structure (**A**) Pressure fluctuation during cavitation growth. (**B**) Density variation (Exclude cavity volume during density calculation). (**C**) Cavitation radius (**D**) Cavitation sphericity index.

The cohesive energy between water molecules always tries to maintain equilibrium water density. Since the gel network is immersed in water, it will not expand during the system expansion. As such, during system expansion, water will initially expand but eventually the system expansion will become proportional to bubble volume expansion or bubble growth. The results are shown in Fig. 3 for X strain rate. It has been found that presence of collagen network requires additional pressure to grow cavitation in the “gel” system when compared with cavitation bubble growth in the water model (Fig. 3A). The density variation follows the same trend as pressure (Fig. 3B). The process, in principle, supports Eq. (2). To discuss the molecular mechanisms in detail, the bubble growth process is divided into three distinct regions (See Fig. 3C).

### Region 1: (0–12 ps)

Here, the growth rate is very slow. Note that we created the cavitation by creating new surface (i.e. by removing a water sphere from the center of the rectangular system). As such, two opposing forces simultaneously act – (i) a favorable expansion force that tends to grow the bubble, and (ii) a surface tension force that tends to shrink the bubble. These two competing forces are strong at the beginning; hence, the velocity of bubble growth is very low. As bubble grows further, the surface tension effect starts to become less dominant. When bubble crosses the threshold pressure, a sharp rise in pressure is observed. At this stage, the velocity of bubble growth is maximum (Fig. 3C).

### Region 2: (12–25 ps)

In this region, there is a sharp rise of negative pressure where the work input by volume expansion overcomes the opposing force due to surface tension. The rise in pressure is more pronounced in water than gel, because gel is nearly three times more viscous than water (see Table 1). We have measured the viscosity of water and gelatin system; the methodology is described in Appendix 2 of supplementary materials. Our study of viscosity measurement is well fitted with the empirical correlation (second order polynomial) of viscosity and concentration, studied by Cumper *et al*.^24^. We have noticed that the magnitude of negative pressure in water near the end of region 2 reduces to its initial value (−20 MPa). The bubble volume to box volume ratio at this region is the highest, therefore, a decrease in tensile pressure is observed. Shortly afterwards, it gradually starts to become stable.

**Table 1:** The input values of surface tension, viscosity initial radius and density.

Model	Surface tension(N/m)	Initial radius of bubble (Å)	Viscosity Pa.s x 10^−3^	Density (g/cm^3^)
Gel	0.0976	95.35	0.684	1.05
Water	0.0557	95.16	0.321	1.02
Water experimental	0.07^42^		0.896^43^	0.99
Simulation Result from literature for TIP3P water	0.0523^33^ at 300 K		0.321^34^ at 298 K	1.002 at 298 K

### Region 3: (25–60 ps)

In this region, bubble growth rate is stable for both the systems. It is observed that pressure fluctuates at higher frequencies in water. The oscillatory nature of pressure fluctuation can be explained from structural dynamics point of view. We have discussed dynamics of bubble in “pressure and cavitation radius damping section” in more details. Moreover, in this region the sphericity index of bubble (Fig. 3D) in gel system deviates from water bubble, that gives us the indication of bubble network interaction.

From the pressure plot comparison shown in Fig. 3A, it is observed that the gel like model requires higher negative cavitation pressure than the water only model. We hypothesize that there are three possible reasons for higher cavitation pressure in gel than water. First, even though the gel network does not initially deform during the application of hydrostatic tension to the gel system, the presence of interfacial attraction between the collagen molecules and water (not present in pure water model) requires higher pressure to grow bubble in the gel system. Second, the bubble growth may require additional force for deforming the gel network. Third, surface tension and viscosity of gels may play additional roles.

**Figure 4:**
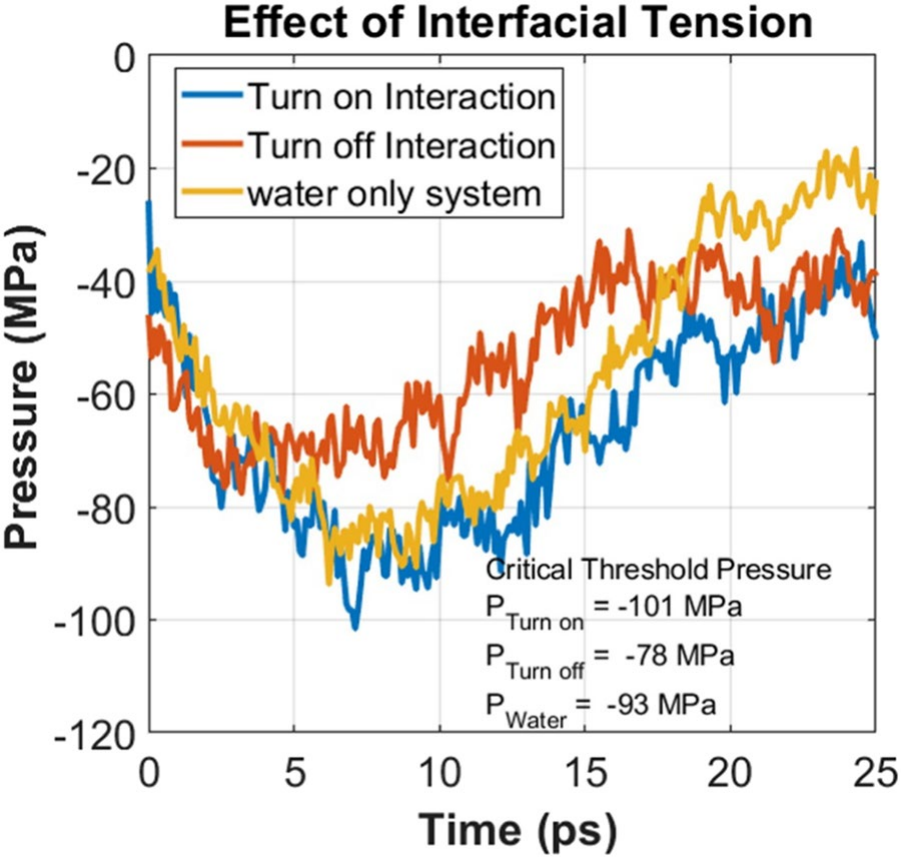
Interfacial tension between water and collagen molecules.

**Figure 5:**
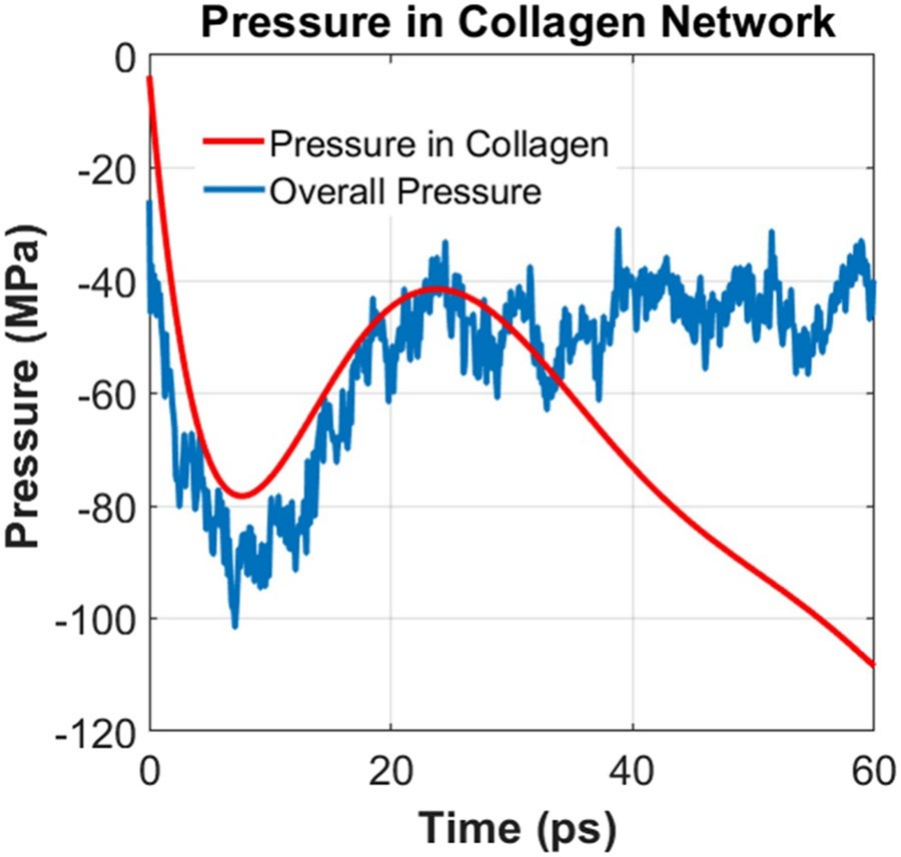
Tensile pressure in the gel network.

To support our first hypothesis we have conducted an additional simulation with the gel model where the interfacial tension between the collagen molecules and water was turned off during the simulation. By comparing the pressure-time relation of this model with the original gel model, the effect of interfacial tension between the collagen molecules and water can be obtained. The results are plotted in Fig. 4. For complete comparison, the pressure history of pure water is also included in Fig. 4. It can be observed that the critical pressure for caviation bubble growth in the pure water is somewhere in between the pressure for caviation bubble growth in the gel with active and inactive interfacial tension. In particular, the critical pressures for these systems (water and gel in inactive interfacial potential) are recorded as −93 MPa and −78 MPa, respectively. On the other hand, when the interfacial tension is active, the critical pressure is about −101 MPa. When the interaction potential between water and collagen molecule is turned off, in principle, the interface region transforms to potential sites for cavitation nucleation. As the hydrostatic tension forces are applied, the threshold pressure goes down because of the absence of interfacial tension between water and collagen molecules. When the interfacial tension is active, the applied hydrostatic tension load is shared by the collagen network and water (Fig. 5). It can be observed from Fig. 5 that although deformation tension is applied to water molecules of gel, at peak pressure point (7.1 ps), the hydrostatic pressure on collagen network reaches to minima (around −78 MPa). As such, it requires higher threshold pressure to grow bubble in the gel system. It can be inferred from the critial pressure comparison that the interfacial tension between water and gel-network contributes has storng influence on the critical pressure for bubble growth.

The mechanisms of bubble growth for gel, gel while turning off interfacial tension and water can be visualized in the supplementary Video-1, 2 and 3, respectively. From the video images, we can observe that the bubble starts to grow uniformly and radially outward. Interestingly, the surface profiles of a bubble appears to be different from a perfect sphere. We argue that a bubble will retain a near-spherical shape when it grows in bulk water. Its shape will deviate from a spherical geometry when it experiences resistance from the surronding gel network. To capture the geometric evolution of the bubble in water and gel, we have compared the real time surface area of our simulated bubble of radius *r* with the surface area of a spherical bubble with the same radius *r*. We refer this ratio as Sphericity Index Ψ. We have calculated Ψ using the following formula^25^:

3 $$\varPsi =\frac{{\pi }^{1/3}{(6{V}_{c})}^{2/3}}{{A}_{c}}$$

where ${{\rm{V}}}_{{\rm{c}}}$ is the volume of the cavity and ${{\rm{A}}}_{{\rm{c}}}$ is the surface area of the cavity. For the perfect round cavitation its value is 1, when it deviates from its spherical shape the sphericity index decreases. Note that at the beginning we have artificially created cavity, which is perfectly round, therefore at the beginning of deformation (0 ps) sphericity index ~1, however when deformation starts the bubble shape sharply deviates from the perfect spherical configuration, both in gel and water. It can be observed from Fig. 3D that the sphericity indices of bubble in water and gel are nearly same up until 25 ps. It implies that the bubble interacts only with the surrpounding water until 25 ps. The strain energy effect of the network comes into play only after 25 ps. It can be noticed in Fig. 3A that pressure reaches its minimum value at around 12 ps. It means, when pressure drops to the minimum value (~12 ps), the bubble surface does not interact with the collagen molecules, as evident from Fig. 3D. It implies that the strain energy in the form of bending, tensile and torsoin of collagen molecule is not resposible for higher cavitation pressure at this regime.

To justify the second hypothesis, we have estimated the pressure on collagen molecules only. We have found that when the bubble starts interacting with the collagen molecules (at 25 ps) to grow furthur, it needs to overcome the resistance arising from the deformation (predominantly bending) of the gel network. It can be observed that (Fig. 5) collagen molecules experiences higher tensile pressure (negative slope) after 25 ps although the tensile pressure in the overall system is less tensile. It can be inferred that as time progresses from 25 ps and onward, the bubble growth mechanism transistions from affine to non affine^26^ implying the network elasticity starts contributing. Appendix 3 of supplementary material shows the elastic properties of collagen molecules at different structural conformations. It can be noticed that the stiffness of the gel-like structure is about 0.53 GPa.

To validate the effect of surface tension on cavitation pressure, we have estimated the surface tension values of gel and water model. We have found that the surface tension of gel is about 0.097 N/m whereas the surface tension of water is 0.056 N/m. Although, the reported experimental surface tension value of gelatin is less than our simulation results, it is known that the surface tension of gelatin is not an static value, rather it decreases with the gelation process^16^. Simulation of gelation process is out of scope of molecular dynamics study, therefore, we assume that the measured surface tension is static during bubble growth and collapse process studied here. Nevertheless, the higher surface tension values in the gel system implies more pressure is required to cavitate in gel (See Appendix 1 of supplementary material).

It is already discussed that in the gel system, the bubble starts to interact with the fibril network after 25 ps. It can be noticed from Fig. 3D that the sphericity index of bubble in water and gel starts to deviate after 25 ps. It implies that the surface profile of bubble in gel is affected by the interaction with the gel network. Since such interaction reduces the sphericity index of the bubble in gel compared to the sphericity index of the bubble in water, higher bubble surface area is created by the bubble in gel than the bubble in water. This is because surface area to volume ratio is minimum for sphere. Any reduction in sphericity index will imply higher surface area when total volume is fixed. As such, an additional energy is required to grow the cavitation bubble in gel. In Wonmo Kang’s work^7^, the shape of a preexisting bubble in gel is predicted as non-spherical. In a way, our simulation at the nanoscale justifies Wonmo Kang’s predictions^7^.

In summary, we can infer that the requirement of higher pressure in growing a cavitaion bubble in gel is primarily associated with the interfacial interactions between the gel and water and the surface tension of gel. The deformation energy of the network does not provide significant influence. However, the presence of the network affects the surface area (i.e. the sphericity index) of the bubble.

### Pressure and cavitation radius damping

It is apparent from the Eqs. (1) or (2) that a stable bubble will maintain its radius if pressure is maintained constant. Without losing generality, it can be inferred from these equations that small fluctuation in pressure will lead to oscillation of bubble radii. For example, for the typical stable bubble radii commonly found in water, which varies between 1 and 100 μm, the natural frequencies are of the order, 5 to 25 kHz^27^. Knowing the frequency of oscillations of bubble in relation to an applied oscillatory pressure has many practical implications. As Brennen explained^27^, if one plans to create cavitation in water using an acoustic pressure field, then the frequencies to most effectively generate a substantial concentration of large cavitation bubbles has to be in this frequency range. Another application where this frequency estimate is useful is in the design of cavitation damage resistant metallic turbine propellers. It is known that to test a metal’s durability against severe cavitation damage, magnetostrictive devices are used to oscillate solid material samples in water (or other liquid) at these critical frequencies (kapp). Although the oscillation of the bubbles produced in this manner may be highly nonlinear, such devices can be very helpful in generating bubble clouds in fluid.

**Figure 6:**
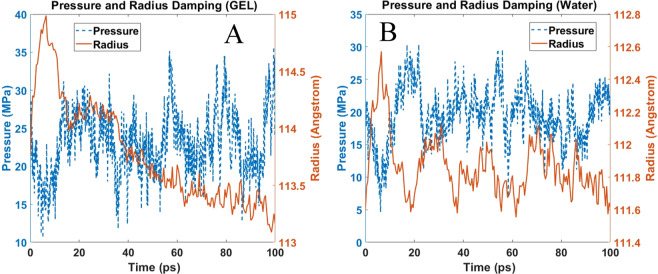
Oscillation of pressure and radius after the growth stage at constant volume (**A**) Gel, (**B**) Water.

In order study this concept further, we studied bubble dynamics at constant system volume. From MD simulation perspective, when the system volume is fixed and a finite temperature prevails in the system, pressure must fluctuate with time due to thermal oscillation. To simulation pressure oscillation in our system, after cavitation growth, we have fixed the volume and determined the pressure oscillation and the natural frequency of cavitation for both the water and gel like models. It is quite interesting to note that pressure fluctuation in the water and gel system is similar (Fig. 6), however presence of collagen dampens the oscillation of the bubble volume in gel like solution. Strasberg (1953)^28^ and Morioka Mikio^29^ shows that for a nonspherical bubble, it is convenient to write a differential equation of the small amplitude, non-condensable pulsing bubble in terms of the volume pulsation v, as shown below:

4 $${\rm{m}}\frac{{{\rm{d}}}^{2}{\rm{V}}}{{{\rm{dt}}}^{2}}+{\rm{b}}\frac{{\rm{dV}}}{{\rm{dt}}}+{\rm{kV}}=0$$

where, m, b, and k are inertial, dissipation and stiffness constant. k, is proportional to inside gas pressure, in our simulation inside gas pressure is very negligible, therefore we can omit third term. Constant b has four components: ${{\rm{b}}}_{{\rm{vis}}}$ = viscous dissipation, $\,{{\rm{b}}}_{{\rm{th}}}$ = thermal dissipation, $\,{{\rm{b}}}_{{\rm{rad}}}$ = radiation dissipation, b_ph_= dissipation due to liquid to vapor phase transformation. For our MD simulation study, only viscous dissipation is largely effective, since simulation is conducted at constant temperature thus thermal dissipation is negligible, there is no radiation, and no or negligible vapor phase inside the bubble.

5 $${\rm{b}}\,\cong {{\rm{b}}}_{{\rm{vis}}}=\,\frac{\mu }{{{\rm{\pi }}{\rm{R}}}^{3}}$$

$${\rm{where}},\,\mu \,{\rm{is}}\,{\rm{the}}\,{\rm{viscosity}}\,{\rm{coefficient}}$$

The natural frequency and damping constant of the bubble are given respectively by

6 $${f}_{n}=\frac{1}{2{\rm{\pi }}}\sqrt{\frac{k}{m}}\sqrt{1-\frac{{\delta }^{2}}{4}}$$

7 $$\delta =b/\sqrt{k/m}$$

The solution of equation Eq. 4 comes out:

8 $$R(t)=C1\times {e}^{\frac{-bt}{m}}+C2$$

C1 and C2 can be calculated from boundary and initial condition. The measured viscosity of gel is almost double than water (See Appendix 2 of Supplementary materials for viscosity calculation), therefore radius damping (Eq. 8) is higher in gel than water. Our simulation results also validate the analytical results (Fig. 6).

A recent publication by Yonatan *et al*.^30^ shows that 20 W% Ballistic gelatin (BG) has 25 times higher ultrasound frequency damping rate than water. However, the damping due to impact force is not that pronounced asserting that a gel like interconnected network structure potentially buffers the stress field.

### Effect of strain rate on bubble growth

**Figure 7:**
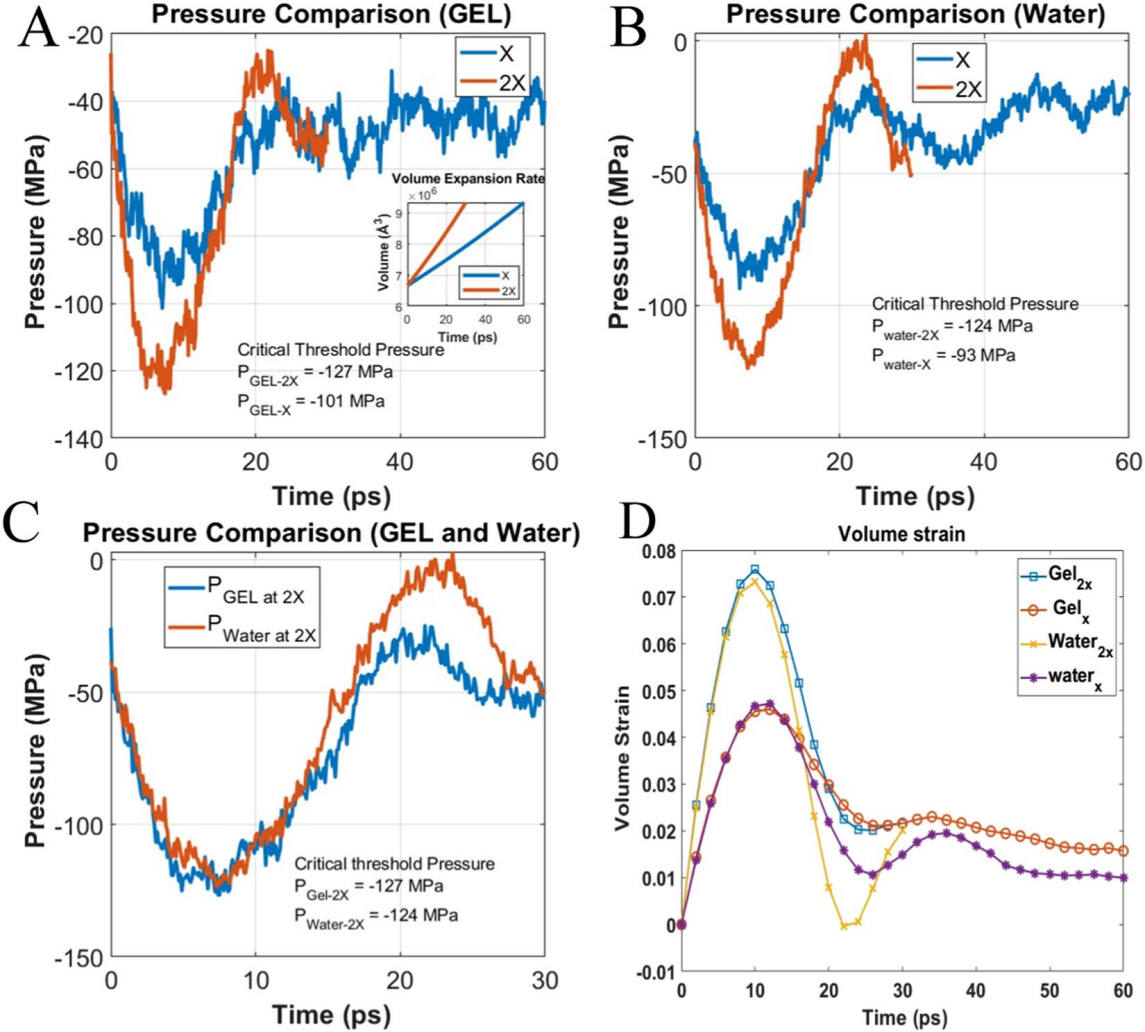
Strain rate effect on cavitation pressure (**A**) Cavitation pressure of gel at different strain rate (**B**) Cavitation pressure of water at different strain rate (**C**) Cavitation pressure comparison between water and gel at higher strain rate (**D**) Volume strain of comparison of gel and water at different strain rate. (Bubble growth at higher strain rate up to 30 ps and in lower strain rate bubble grows up to 60 ps).

**Figure 8:**
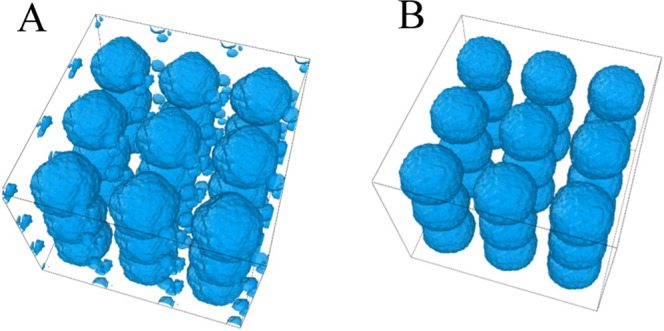
Cavitation at the end of bubble growth at 2X strain rate of (**A**) Gel, (**B**) Water. (For better visualization of cavity, all the atoms are deleted from the system, 3 fold periodic image in each axis) (Image captured by Ovito [‘https://www.ovito.org/’ version 3.0.0-dev]^27^).

The effect of strain rate on the cavitation threshold pressure is shown in Fig. 7. It has been found that bubbles at higher strain rate requires additional pressure to grow. Stan *et al*.^12^ studied cavitation growth by imposing X ray induced shock wave and found that decompression time reduction from 20 ns to 2 ns reduce the cavitation pressure requirement from −30 MPa to below −100 MPa. Arvengas *et al*.^19^ showed that at higher acoustic frequency the cavitation formation in water requires minimum negative pressure. Our study also complies with the experimental observation. We have expanded the system volume to grow bubble for 60 ps and 30 ps at X and 2X hydrostatic strain rate for the same percentage of volume expansion in water and gel system and observed higher negative pressure required for the bubble growth at higher strain rate. Figure 7 shows the result of deformation of the gel and water system at different strain rates. It has been found that increasing the strain rate decrease the minimum threshold pressure 26 MPa in gel (Fig. 7A). On the other hand, in the water system it decreases 31 MPa (Fig. 7B). The effect of strain rate in gel and water is quite similar. While comparing the threshold pressure of Gel and Water at 2X strain rate (Fig. 7C) the difference is only 3 MPa, whereas at lower strain rate the difference is 8 MPa, despite the fact of higher cavitation pressure in Gel. Moreover, some additional nucleation site is observed in gel system at higher strain rate. Figure 8A shows the homogenous nucleation of bubble in gel system. Instantaneous formation of very tiny bubbles can be noticed in gel (Fig. 8A) that are absent in water (Fig. 8B).

Cavitation pressure requires some time to propagate in the gel system (due to higher damping see the previous section) at a higher strain rate until the local pressure reached the homogenous cavitation pressure, and homogenous cavitation initiates (video-4). Time lapse in gel system is due to viscous effect. Figure 7D shows the strain at different strain rates for both the gel and water models. Volume strain is maximum at a peak pressure for both the systems. The gel strain is higher at higher strain rate, because the peak pressure at localized region surpasses the homogenous nucleation threshold pressure (~150 MPa)^31^ of water. Moreover, deformation is applied only in water which caused cavitation to form adjacent to gel fibril, due to higher rigidity of gel fibril it is reasonable to assume that deformation mainly applied to the water molecules at that time scale and in this region I.

Minimum threshold pressure reached for water at ~6.2 ps and ~7.4 ps for X and 2X strain rates, respectively, whereas for gel it reached at 7.1 ps and 7.5 ps for the corresponding strain rates. Both the systems are expanded at the same rate and cavitation radius is almost same at respective times (see Fig. 3C). The delayed cavitation threshold pressure for gel at both strain rate is due to the higher viscosity of gel. Interestingly, at lower strain rate of gel minimum threshold pressure reached 1 ps after the water at similar dynamic condition, however at 2X strain rate critical pressure reached at the same time, which gives us the impression that at higher strain rate effect of network is insignificant to determine the critical threshold pressure.

### Cavitation bubble collapse

**Figure 9:**
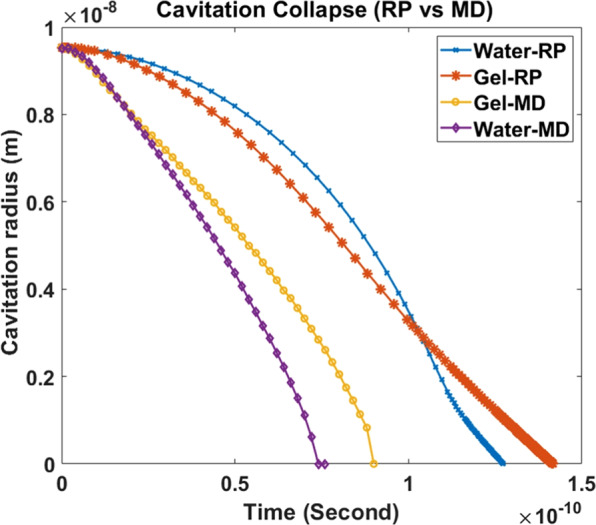
Bubble collapse time estimation from RP equation and compare with MD result.

Here, we have investigated the simulated bubble collapse mechanisms. The changes of bubble radii in gel and water with time have been recorded and plotted, as shown in Fig. 9. The simulation results are also compared with the well-known Rayleigh-Plesset (RP) Equation^32^, as shown in Eq. 9.

9 $$-\frac{({P}_{ext}-{P}_{b})}{\rho }=R\frac{{d}^{2}R}{d{t}^{2}}+\frac{3}{2}{\left(\frac{dR}{dt}\right)}^{2}+\,\frac{4\eta }{\rho R}\frac{dR}{dT}+\frac{2\sigma }{\rho R}$$

where, R is the bubble radius, ${{\rm{P}}}_{{\rm{ext}}}$ the pressure in the liquid, ${{\rm{P}}}_{{\rm{b}}}$ the pressure inside the bubble, and ρ, η and σ are, respectively, the density, viscosity, and surface tension of the liquid. By solving this equation, the radius vs time data is obtained and plotted (Fig. 9). It can be observed that the bubble collapse time obtained from the MD simulation is different from the collapse time predicted by the RP equation. There are few reasons for such deviation. The RP equation assumes bubbles are perfectly spherical in shape, however we have found that bubbles are nonspherical in nature (Fig. 3D). Since the stability of a nonspherical bubble is lower than a spherical one, the bubble in the MD study receives more driving force (to minimize energy) to collapse. In the RP equation, we have ignored the thermal term, the bubble surface and far field temperature is considered constant. In addition, the liquid density, surface tension and dynamic viscosity are considered constant^27^ in the RP equation. However, based on our observation from the simulation data, density does not remain constant over time. We observed that (Fig. 3B) at the peak pressure, the liquid (water) density starts to decrease from the initial density. Since the bubble is nanosized and hardly a few water molecules in vapor state can exist during collapse to put significant pressure on the bubble surface, it is reasonable to assume that ${{\rm{P}}}_{{\rm{b}}}$ is negligible^11^. We have estimated the dynamic viscosity by Green-Kubo method (see Appendix 2 of supplementary materials) and the surface tension is measured following the protocol described by Bhatt *et al*.^33^. We acknowledge that the viscosity of water obtained from our simulation is significantly lower than the experimentally measured one^34^. This is possibly because of the TIP3P water model we used in our simulation^34^. It is interesting to note that in the solution to the RP equation for water and gel (Fig. 9), the velocity is retarded just before the collapse. At constant far field pressure and zero vapor pressure, the RP equation has only two competing variables, the viscous term ($\frac{4{\rm{\eta }}}{{\rm{\rho }}{\rm{R}}}\frac{{\rm{dR}}}{{\rm{dT}}}$) and the surface tension term ($\frac{2{\rm{\sigma }}}{{\rm{\rho }}{\rm{R}}}$). Instantaneous velocity of the bubble is multiplied in the viscous term but it is absent in the surface tension term. Since the velocity increases with time, at some point the viscous term starts to dominate to retard the collapse.

**Figure 10:**
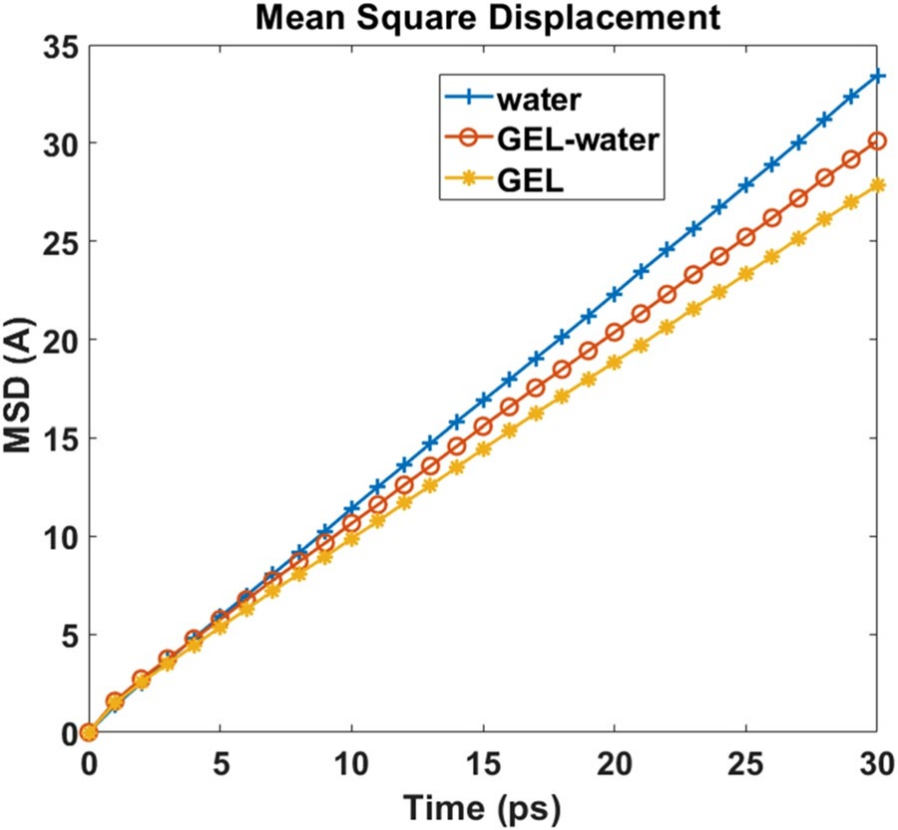
MSD of Water and gel model, for comparison MSD of water of gel model is also shown.

When the collapse time of bubbles in gel and water is compared, it can be observed that the gel model requires more time to collapse than pure water. We argue that the difference in viscosities is playing the major role for the delayed collapse in the gel system. In principle, viscosity is inversely proportional to molecular mobility or diffusion. To compare the diffusion of different molecules in the water and gel system, we have estimated the Mean Square Displacement (MSD) of gel, water and water of the gel (excluding the collagen network) as shown in Fig. 10. For a 3D system, diffusion coefficient is one sixth of MSD. It can be observed from Fig. 10 that the MSD of water molecule in water system is higher than the gel system justifying the delayed collapse time of gel. Due to hygroscopic^35^ nature of collagen molecule, interfacial water are bound to hydrophilic residues of collagen molecules. For comparison, MSD of water of gel model is also calculated. Interestingly, in the gel model the water losses mobility due to the presence of collagen molecule.

## Methodology

**Figure 11:**
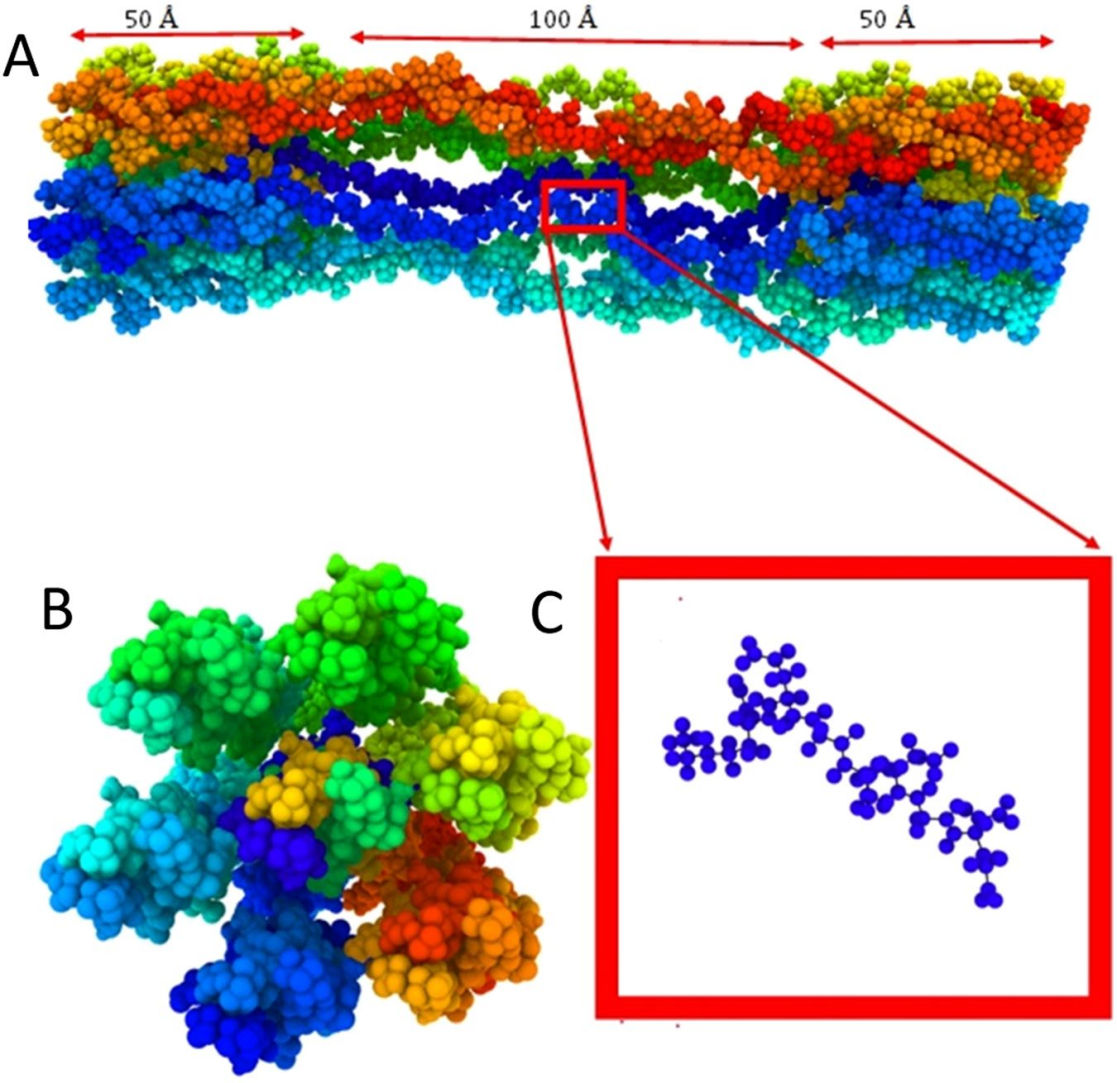
Gel Model (**A**) Full model of gel fibril (**B**) Cross sectional view of fibril (**C**) Individual coil profile. (Image captured by Ovito [‘https://www.ovito.org/’ version 3.0.0-dev]^32^).

Collagen structure (Protein Data Bank identification code 3HR2) used for this study is obtained from the author of this work^36^. Homology modelling approach is adopted to build the protein model using modeler software. The collagen triple helix is 3000 Å in length and ~15 Å in diameter. We have cut only 200 Å from the full length collagen simply by editing the PDB file. We have built an hexagonal array of microfibril like structure by placing seven triple helix at six corner of hexagon and one in the center point of hexagon by using UCSF chimera protein visualization software (https://www.cgl.ucsf.edu/chimera/, version 1.13.1)^37^. To replicate a hydrogel like structure the microfibril structure is further edited to remove few alpha 1 and alpha 2 chains from the triple helix. The mid 100 Å along the length direction is more of like coil and 50 Å from both ends is more like the micro-fibril like structure (Fig. 11). The cavitation simulation model is designed by simply copying the gel like microfibril model at different position of the box (211 × 211 × 165). While placing the gel like model some rotation and translation is applied by UCSF chimera. All models are solvated by TIP3P water using CHARMM-GUI^38^ Solvator. Two models are created of same box dimensions, where percentage of collagen is 10.4% w/w, and 0% w/w respectively (shown in Fig. 2).

The interatomic potential has been adopted from the CHARMM36 force field^39^ and the simulation is carried out by LAMMPS^40^ on STAMPEDE2 super computer. For visualization of LAMMPS trajectory file, we have used OVITO [‘https://www.ovito.org/’ version 3.0.0-dev]^32^ visualization software. We have used MATLAB for data analysis.

All models are equilibrated for around 200 ps until the potential energy became stable in isothermal-isobaric (NPT) ensemble, where Nose-Hoover thermostat is applied to equilibrate the temperature to 310 K with a damping constant 100 fs, and Nose-Hoover barostat is applied to equilibrate pressure to zero (ATM) with a damping constant of 1000 fs. Lenard jones pair style is used with an inner cutoff radius 8 and outer cutoff radius 10. Long range coulombic interactions are computed by pppm style which invokes a particle-particle particle-mesh solver which maps atom charge to a 3d mesh, uses 3d FFTs to solve Poisson’s equation on the mesh, then interpolates electric fields on the mesh points back to the atoms. Periodic boundary condition is applied at all three directions.

For cavitation study, isostatic pressure is applied by applying constant volume expansion rate. Rate dependency on cavitation pressure is examined by application of volume expansion at two different strain rates (X and 2X). Bubble radius is measured by ovito “construct surface mesh” tool at different timestep. We have restricted the movement of the end chain c-alpha atom during the cavitation study and uniform deformation is applied to the water molecules only. Stress/atom is calculated to measure the pressure. Stress/atom gives the value of (stress x volume) in each direction for each atom, dividing by volume of each atom gives us the exact amount of virial stress. Global pressure, P = (Pxx + Pyy + Pzz)/(3 × V), where Pxx, Pyy and Pzz is the summation of stress/atom value for all atoms in x, y, and z direction respectively and V is the summation of each atom’s volume in the system. For volume measurement, voronoi cell is created around each atom using Voro++ package^41^ which is compiled in LAMMPS.

## Conclusion

We have studied the nanobubble growth and collapse mechanisms in pure water and gel model. Due to computation limitation the radius of the bubble is limited to 5 nm only. The following conclusions can be drawn from our current study:

- The results suggest that cavitation bubble requires higher pressure to grow in gel solution. The additional pressure is associated with the energies to overcome interfacial tension of water and gel, bending stiffness of gel like fibril, and surface tension of the solution.
- At higher strain rates, the gel like structure invokes homogenous nucleation despite the presence of large bubble, which suggests that pressure propagation rate in gel is lower than that in water. In other words, the presence of gel microfibril facilitates homogenous nucleation.
- Cavitation collapse time is dominated by viscosity of medium. Surface tension plays insignificant role.

Thermodynamics of bubble growth needs detailed analysis in the free energy perspective. Quantification of cohesive force between gel fibril and water, and strain energy absorption by fibril may give us the correlation between threshold pressure difference of water and gel system during bubble growth. A detail study is underway and will appear in our future publications.

## Supplementary information

Supplementary Information.

Supplementary Information2.

Supplementary Information3.

Supplementary Information4.

Supplementary Information5.

Supplementary Information6.
