# Supplementary material for: On the Molecular Level Cavitation in Soft Gelatin Hydrogel
Source: Sci Rep. 2020 Jun 15;10:9635. doi: 10.1038/s41598-020-66591-9 (PMC7295970; doi:10.1038/s41598-020-66591-9)
Supplement: Supplementary file 1 — Supplementary Information. [file 41598_2020_66591_MOESM1_ESM.docx]

Video-1: The simulation video shows the growth of cavitation in gel like solution at lower strain rate (X)

Video-2: The simulation video shows the growth of cavitation in gel like solution at lower strain rate (X), while turning off the interfacial interaction.

Video-3: The simulation video shows the growth of cavitation in water at lower strain rate (X)

Video-4: The simulation video shows the growth of cavitation in gel like solution at higher strain rate (2X)
