# Supplementary material for: On the Molecular Level Cavitation in Soft Gelatin Hydrogel
Source: Sci Rep. 2020 Jun 15;10:9635. doi: 10.1038/s41598-020-66591-9 (PMC7295970; doi:10.1038/s41598-020-66591-9)
Supplement: Supplementary file 2 — Supplementary Information2. [file 41598_2020_66591_MOESM2_ESM.docx]

**Supplementary material**

**On the molecular level cavitation in soft gelatin hydrogel**

KAH Al Mahmud^a^, Fuad Hasan^a^, Md Ishak Khan^a^ , Ashfaq Adnan^a,^ ^[[1]](#footnote-1)^

^a^Mechanical and Aerospace Engineering, University of Texas at Arlington

# **Appendix 1: Surface tension measurement**

We have measured the surface tension following the protocol stated by Vega el at. ^1^. Our box size is 211× 211× 165. Open surface is created in the z direction. For a subcritical temperature, this setup is expected to stabilize two planar vapor-liquid interfaces perpendicular to the z axis of the simulation cell. Molecular dynamics simulations are performed using LAMMPS to generate the molecular trajectories using a time step of 1 fs. The temperature was kept constant by using a Nose-Hoover thermostat with a damping constant of 1 ps. The inhomogeneous system is first allowed to equilibrate over 300 ps, and running averages are then collected over an additional run of around 1.5 ns depending on the thermodynamic conditions. The LJ part of the potential is truncated at 8 Å and a switching function is used between 8 and 10 Å. Long range columbic potential is calculated using pppm (particle-particle particle-mesh) solver, which maps atom charge to a 3d mesh, uses 3d FFTs to solve Poisson’s equation on the mesh, then interpolates electric fields on the mesh points back to the atoms. The diagonal component of pressure is used to measure the surface tension. Here normal component of pressure is Pzz, whereas tangential component of pressure is arithmetic mean of Pxx and Pyy.

Surface tension, γ= $\int_{\text{∞}}^{\text{∞}} \text{d}z$ ($\text{P}_{\text{N}}$(Z) - $\text{P}_{\text{T}}$(Z))

Where,

P_N_ (Z) = Normal components of pressure tensor,

P_T_ (Z) = Tangential components of pressure tensor


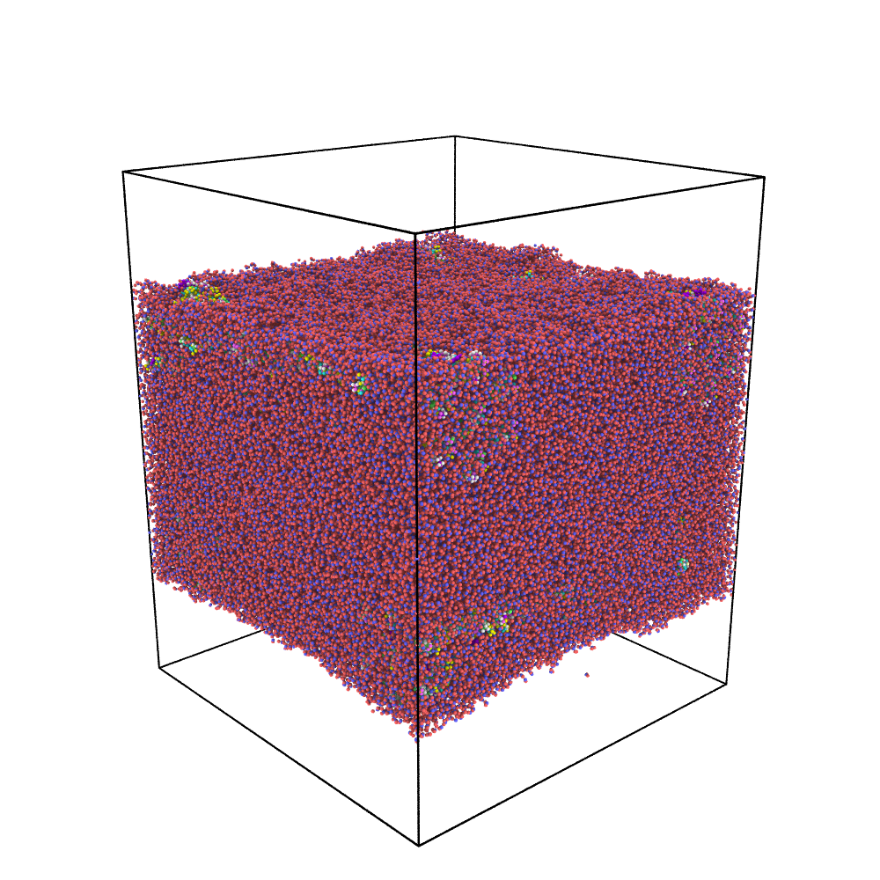


Z

Figure 1‑A simulation box for surface tension measurement(Image captured by ovito ^2^ [‘<https://www.ovito.org/>’ version 3.0.0-dev])

# **Appendix 2: Viscosity Measurement**

We have measured dynamic viscosity by Green Kubo technique ^3^ of gel like structure and water in LAMMPS. The detail scripting of viscosity measurement is stated in LAMMPS ^4^. We have solved the following equation for viscosity measurement. We have used NVT ensemble for viscosity calculation, where temperature was fixed at 310K with a damping constant 1ps. To get appropriate viscosity value simulation was run for 100 ps. LJ cut off was fixed at 8 Å and outer cutoff was 10 Å. Electrostatic force was calculated using PPPM technique. Figure 2 shows the initial simulation box of viscosity calculation, where box size 211 Å × 211 Å × 165Å.

Dynamic viscosity, $\text{η}$= $\frac{\text{V}}{\text{k}_{\text{B}}\text{T}}$ $\int_{\text{0}}^{\text{∞}} \text{dt}$ ($\text{P}_{\text{xy}}$ (0)$\text{P}_{\text{xy}}$(t))

Where,

V= volume of the particle system

T= is a temperature, 310K

$\text{k}_{\text{B}}$= Boltzmann constant

$\text{P}_{\text{xy}}$= off-diagonal element of the stress tensor


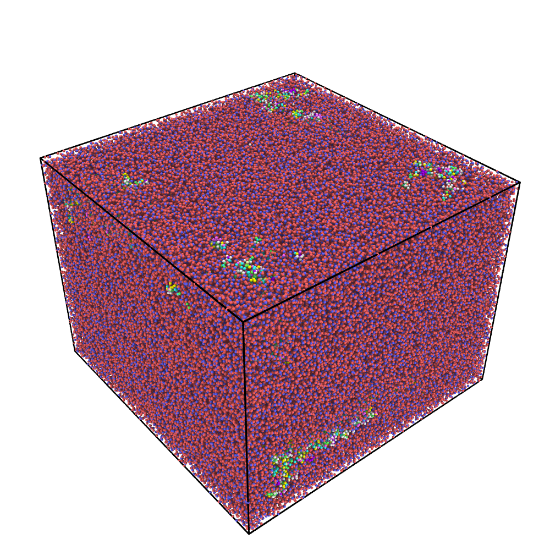


Figure 2‑A Simulation box for dynamic viscosity measurement (Image captured by ovito ^2^ [‘<https://www.ovito.org/>’ version 3.0.0-dev])

# **Appendix 3: Mechanical Properties of Collagen Molecules**

We have conducted the mechanical property evaluation of collagen by steered molecular dynamics (SMD) simulation at different scale such as single coil, double coil, triple helix and gel like structure (*Figure 3‑A* ). Each structure is 100 Å in length except the gel like structure. To maintain constant strain rate gel like structure is pulled at twice as much as the as the triple helix pulling velocity. In SMD approach constant velocity is applied for all cases, however for coil, spring constant chosen to be K=5 Kcal/mol. Å ^2^ and for other models 20 Kcal/mol. Å ^2^ is chosen (*Figure 3‑A* ). All the SMD simulation is carried out at NVT ensemble at a strain rate of 1 x 10^9^ s^-1^.


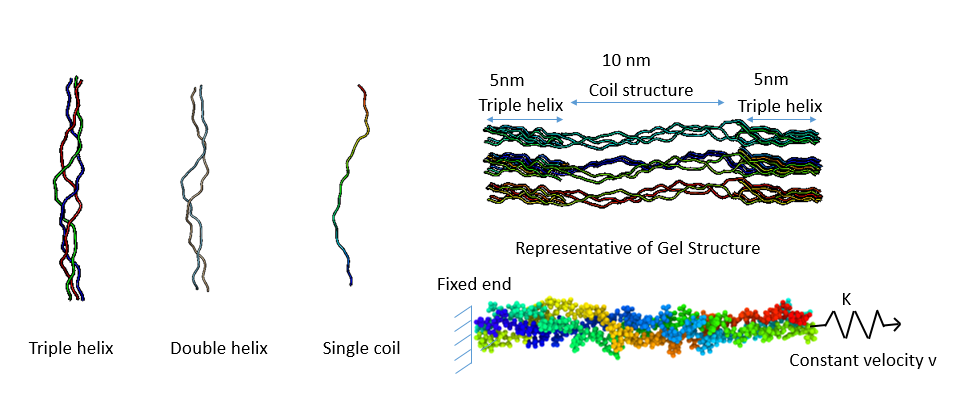


C

B

A

Figure3‑A Collagen profile at different scale (A) collagen segmented profile (Triple helix, double helix, coil) (B) Gel like structure for our model to study cavitation (gel like fibril is modeled using UCSF chimera [‘<https://www.cgl.ucsf.edu/chimera/> ‘ version 1.13.1]^5^) (C) SMD pulling approach for strength determination (Image captured by ovito ^2^ [‘<https://www.ovito.org/>’ version 3.0.0-dev])

To determine the mechanical properties of collagen molecules that can be later used in the cavitation study of gel, a set of stretching simulations are conducted. For the stretching simulation, we have tested triple helix, double helix, single coil and gel like fiber configurations at a strain rate of 1 × 10^9^ s^-1^. The rate is chosen based on cavitation collapsing strain rate ^6^. After equilibration, a uniform velocity field is applied along the longitudinal direction. During equilibration, almost all the models shrink to some extent, initial length is considered the length of the equilibrated structure. The gel like structure and triple helix show maximum and minimum shrinkage respectively during equilibration. The developed force is evaluated by MD simulation study (SMD approach), where constant velocity is applied from one end and other end is fixed. In the fix end, one alpha carbon atom from each chain is restricted by applying zero force and velocity. The schematic is shown in the inset picture of Figure 4-A. Since collagen chains are not uniform in cross-section along their length, an initial “average” cross sectional area of the simulated molecules is estimated by taking the average of the projected cross sectional areas along the longitudinal direction at 10 different positions. The projected cross section area image from those positions are taken by Ovito visualization software ^2^. Later, those images were analyzed by Fiji ImageJ ^7^ image processing software to estimate the average surface area. To calculate developed tensile stress, we have divided the developed tensile force with this initial “average” cross sectional area.

The stress-strain results are reported in Figure 4-A. It can be observed that the helical arrangements of the coils in the triple helix system provides highest resistance to deformation. For this, this configuration has higher stiffness compared to other configurations tested. The single coil structure, on the other hand, has the lowest stiffness. The double helix configuration has intermediate stiffness. Since it is a metastable structure, part of the initial configuration of the double helix evolves to helical configuration during simulation. This transformation is responsible for the higher stiffness in double helix system compared to the single coil configuration. In the gelatin, all three types of configurations are present. During deformation, the gel like structure behaves more like a coil structure, except at the beginning of pulling when it shows more stiffer than the coil. The two ends of the gel-like structure (which is pulled and fixed) is configured more like a triple helix. As such, the end region of the gel deforms like a triple helix. When the stress is transferred to the single-coil region of the gel-like system, it starts to behave like the coil structure.


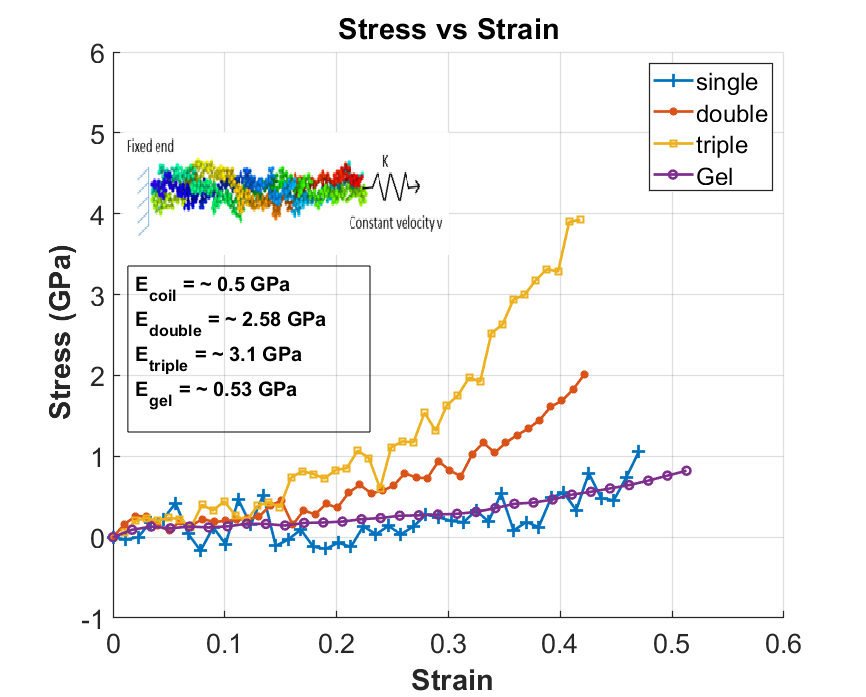


**Figure 4-A** stress strain comparison of coil, double helix, triple helix and Gel like structure

# **Appendix 4: Important Properties of gel**

**Surface Tension:** Surface tension depends on the thermal history of gelatin, percentage of triple helix content in gel, concentration^8^, and gelation time ^9^. In general, quenching after full denaturation of gelatin makes it harder for the coil to form triple helix. As such, the process increases surface tension because coil has higher surface energy than triple helix. Surface tension also depends on gelation time because in principle, the triple helix content increases as the time progresses, irrespective of different concentration of solution. Since higher triple helix content reduces overall surface energy, the surface tension of gel decreases as the gelation time increases. ^10^

**Viscosity:** Viscosity of gelatin is an important parameter which influences the mechanics of gel solution in different conditions. Viscosity of gel generally varies exponentially with gel concentration; as such, at lower concentration (1 to 3%), viscosity of gel does not change significantly. However, at concentration of 5% and above, viscosity of gel rises sharply with concentration increase. In general, viscosity of gel is significantly higher than water. Such differences come from different intermolecular interaction^11^ including coulombic forces between oppositely charged ions (presumably mainly -COO- and ${-NH}_{3}$+), ion-dipole forces (e.g. -COO- on one chain and,${-NH}_{2}$ on the other ; or ${-NH}_{3}+$ and -COOH), hydrogen bonding between >C=0 and H-N<, london forces, particularly between the non-polar side-chains, mechanical entanglements between the long chains as found with non-polar polymers in ideal solvents.

**Rigidity:** Empirical correlation of rigidity of the gelatin solution at the different concentrations, average molecular weight and temperature has been found ((Ferry and Eldridge 1949) ^12^) :

$n^{1/2}/c$ = 1.22 х ${10}^{-4}(M_{w}-3.1 х {10}^{10} e^{-7900/RT}$)

Where, n is the modulus of rigidity, C is the concentration, $M_{w}$ is the average molecular weight of gel in solution. In general variation of rigidity and viscosity depends on the same factors except some fundamental differences. All the ionized side-chains of gel molecules will contribute to the viscosity (the system being mobile, over a sufficient time all possible types of side-chain contact will occur, and the observed effect will thus be an average one) whereas the rigidity will be determined solely by those few side-chains that actually form intermolecular salt bridges and possess lowest energy consistent with steric restrictions^11^.

References:

1. Vega, C. & De Miguel, E. Surface tension of the most popular models of water by using the test-area simulation method. *J. Chem. Phys.* **126**, 154707 (2007).

2. Stukowski, A. Visualization and analysis of atomistic simulation data with OVITO–the Open Visualization Tool. *Model. Simul. Mater. Sci. Eng.* **18**, 15012 (2009).

3. Ogawa, H., Shiraishi, Y., Kawamura, K. & Yokokawa, T. Molecular dynamics study on the shear viscosity of molten Na2O·2SiO2. *J. Non. Cryst. Solids* **119**, 151–158 (1990).

4. Plimpton, S. Fast parallel algorithms for short-range molecular dynamics. *J. Comput. Phys.* **117**, 1–19 (1995).

5. Pettersen, E. F. *et al.* UCSF Chimera—a visualization system for exploratory research and analysis. *J. Comput. Chem.* **25**, 1605–1612 (2004).

6. Wu, Y.-T. & Adnan, A. Damage and Failure of Axonal Microtubule under Extreme High Strain Rate: An In-Silico Molecular Dynamics Study. *Sci. Rep.* **8**, 12260 (2018).

7. Schindelin, J. *et al.* Fiji: an open-source platform for biological-image analysis. *Nat. Methods* **9**, 676 (2012).

8. Yang, H. & Wang, Y. Effects of concentration on nanostructural images and physical properties of gelatin from channel catfish skins. *Food Hydrocoll.* **23**, 577–584 (2009).

9. Mackie, A. R., Gunning, A. P., Ridout, M. J. & Morris, V. J. Gelation of gelatin observation in the bulk and at the air‐water interface. *Biopolym. Orig. Res. Biomol.* **46**, 245–252 (1998).

10. Estrada, J. B., Barajas, C., Henann, D. L., Johnsen, E. & Franck, C. High strain-rate soft material characterization via inertial cavitation. *J. Mech. Phys. Solids* **112**, 291–317 (2018).

11. Cumper, C. W. N. & Alexander, A. E. The viscosity and rigidity of gelatin in concentrated aqueous systems. II. Rigidity. *Aust. J. Chem.* **5**, 153–159 (1952).

12. Ferry, I. J. D. & Eldridge, J. E. Studies of the Cross-Linking Process in Gelatin Gels. *J. Phys. Chem.* **53**, 184–196 (1949).

1. Corresponding Author: Dr. Ashfaq Adnan

   Email: aadnan@uta.edu [↑](#footnote-ref-1)
